# The Relationship between Daily Physical Activity, Psychological Factors, and Vegetative Symptoms in Women with Fibromyalgia: A Cross-Sectional Observational Study

**DOI:** 10.3390/ijerph191811610

**Published:** 2022-09-15

**Authors:** Santiago Navarro-Ledesma, Leo Pruimboom, Enrique Lluch, Lirios Dueñas, Silvia Mena-Del Horno, Ana Gonzalez-Muñoz

**Affiliations:** 1 Department of Physiotherapy, Faculty of Health Sciences, Campus of Melilla, University of Granada, Querol Street 5, 52004 Melilla, Spain; 2 University Chair in Clinical Psychoneuroimmunology (University of Granada and PNI Europe), Querol Street 5, 52004 Melilla, Spain; 3 PNI Europe, 2518 JP The Hague, The Netherlands; 4 Department of Physiotherapy, Faculty of Physiotherapy, University of Valencia, Street de Gascó Oliag, 5, 46010 Valencia, Spain; 5 Physiotherapy in Motion, Multi-Specialty Research Group (PTinMOTION), Department of Physiotherapy, University of Valencia, 46010 Valencia, Spain; 6 Pain in Motion International Research Group, Vrije Universiteit Brussel, 1050 Brussels, Belgium; 7 Clinica Ana Gonzalez, Avenida Hernan Nuñez de Toledo 6, 29018 Malaga, Spain

**Keywords:** fibromyalgia, chronic pain, autonomic nervous system, psychological, physical activity

## Abstract

Nowadays, there is evidence that relates the amount of physical activity, as well as the impact of psychological factors, to the intensity of symptoms present in patients with fibromyalgia (FM). However, there are no studies which correlate the level of association of physical activity, psychological factors and vegetative symptoms in the FM population. The study has a cross-sectional observational design with 41 participants being recruited from a private clinic and rehabilitation service. The Autonomic Symptom Profile (Compass-31) to assess vegetative symptoms, the GODIN questionnaire to evaluate the level of leisure activity, and the pain catastrophizing scale, Tampa Kinesiophobia Scale and Self-Efficacy Scale to assess psychological factors, were used. A low and significant level of association was found between pain catastrophizing (PCS) and Kinesiophobia (r = 0.398; *p* < 0.01), as well as with catastrophizing and vegetative symptoms (r = 0.428; *p* < 0.05). Furthermore, a low and significant level of association was also found between self-efficacy and vegetative symptoms (r = 0.397; *p* < 0.05). No association was found between the level of daily physical activity (measured by the Godin Leisure questionnaire) and vegetative symptoms, nor with any psychological factor studied. There is an association between vegetative symptoms and psychological factors. Nevertheless, more research which takes other factors into account, such as lifestyle and nutritional, is needed.

## 1. Introduction

Fibromyalgia (FM) is defined as a multifactorial chronic disease of unknown origin with generalised pain, fatigue, cognitive disturbances, anxiety and degenerative or inflammatory behavioural disturbances [1,2,3]. The presence of a catastrophic event, physical trauma or emotional trauma are potential triggers for fibromyalgia [4]. The American College of Rheumatology has established distinct criteria for its classification, presenting different diagnostic factors between this syndrome and other rheumatological and pain disturbances, which include the presence of generalized pain which lasts for three months, a digital pressure of 4 kg which causes the sensation of pain and widespread pain with hypersensitivity measured using a map of the characteristic musculoskeletal points in the diagnosis of FM [1,5].

Fibromyalgia affects between 0.2% and 6.6% of the world’s population, thereby making it one of the greatest problems in the general population and the most frequent cause of chronic and diffuse musculoskeletal pain [6]. It is more common in middle-aged adults, with a range of 0.5% to 5% in the general population and up to 17.5% in a clinical setting [7,8].

Furthermore, the presence of FM implies a reduction in the quality of life in those patients who have it [8,9], and other syndromes such as irritable bowel syndrome or chronic fatigue syndrome can be up to 3 times more prevalent in patients with fibromyalgia than in the rest of the population [3]. Nevertheless, the early detection of FM, not to mention the absence of sleep disturbances in this clinical picture, have been shown to lead to a more favourable outcome in those patients with this condition. 

Although numerous signs and symptoms present in FM are already known, this is not the case for the underlying mechanisms. Presently, FM is considered to be a central sensitisation syndrome and the exaggerated and increased pain responses can be explained by changes in neurotransmitters, in addition to abnormalities in the ascending and descending pain pathways, which involves the hypothalamic-pituitary axis (HPA) [10].

Regarding the vegetative nervous system, patients with fibromyalgia are found to have a common anomaly which is postural orthostatic tachycardia. Additionally, these patients show a more acute sympathetic nervous response to stimuli such as cold, auditory stimuli or mental stress. This reaction is seen to generate a greater heart rate increase in patients with FM, which may be mainly due to pain [4]. Furthermore, the vegetative change can also affect the gynaecological area. In this context, there are higher rates of gastrointestinal and gynaecological dysfunctions and pathologies in FM patients compared to healthy patients [11]. Specifically, women with FM have 50% more pelvic floor symptoms compared to women who do not have FM. These patients usually experience pain, urinary and colorectal dysfunction and/or pelvic organ prolapse [12], and this may be explained by altered psychological factors, such as fear or a threatening feeling, which directly alter the so-called Emotional motor system [13].

In addition to psychological factors, there is evidence that relates the amount of physical activity on the intensity of symptoms present in patients with FM. However, there are no studies which correlate the level of association of physical activity, psychological factors and vegetative symptoms in the same FM population. The undertaking of this study will help in understanding the relationship between these factors and help to elaborate future studies that will take these components into account.

The objective of this study is to analyze the level of association between physical activity, psychological factors and the presence of vegetative symptoms in patients with FM.

## 2. Methodology

### 2.1. Design

The study has a cross-sectional observational design.

Participants were recruited from a private healthcare centre in Malaga, Spain. Potential referrals were informed about the trial via formal meetings and trial information sheets. This study is reported in line with the standard protocol elements: Statement of Recommendations for Intervention Trials (STROBE).

This study protocol has received ethical approval by the Ethics Committee of Human Research at the University of Granada, Spain (1044/CEIH/2020). All participants will accept and sign an informed consent form before beginning the study.

### 2.2. Participants

**Figure 1:**
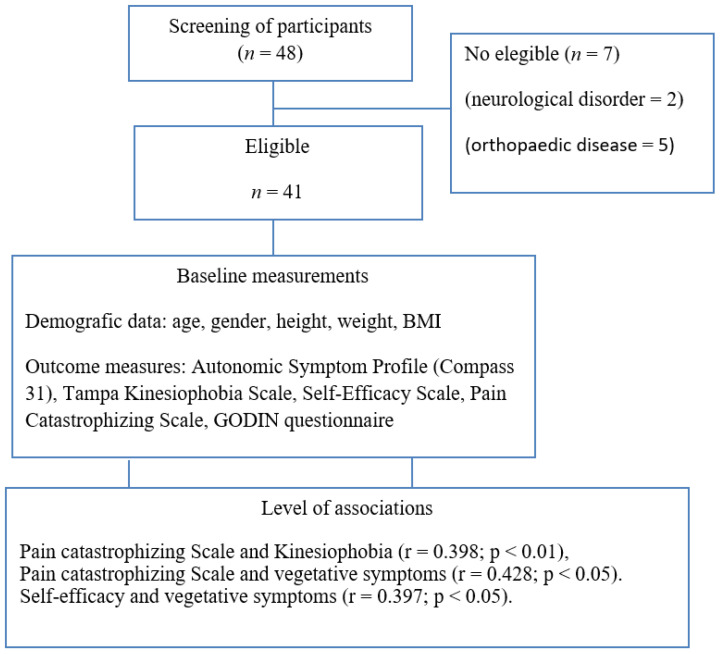
Flow diagram of participants.

A total of 48 women with FM participated in the study, and a final number of 41 were included after assessing inclusion and exclusion criteria (see Figure 1). Participants were recruited from a private clinic and rehabilitation service in Malaga, Spain. The physiotherapist, who was in contact with the participants for the recruitment process, provided them with information about the study and eligibility criteria. All participants signed a consent form to participate in the study. The participants were evaluated by the physiotherapist to determine whether they met the following inclusion and exclusion criteria:

### 2.3. Inclusion Criteria

1. Aged between 18 and 64 years; 

2. Diagnosis of FM by a rheumatologist according to the ACR classification criteria (modified 2010/2011). To diagnose fibromyalgia in adults, it is necessary that all the following criteria be met: presence of generalised pain i.e., in at least 4 of 5 regions, present symptoms lasting at least 3 months at the same intensity, symptom severity score (SSS) ≥ 5 and widespread pain index (WPI) ≥ 7, or SSS score ≥ 9 and WPI between 4 and 6, and a diagnosis of fibromyalgia does not exclude the presence of other illnesses and is valid irrespective of other diseases.

### 2.4. Exclusion Criteria

The presence of any inflammatory, neurological or orthopaedic disease which may alter balance, hearing and vision, or cognitive impairment which may affect the ability to answer questions. In addition, fascial muscle disturbances such as trigger points, myofascial pain syndrome and neck pain.

### 2.5. Primary Outcomes

#### 2.5.1. The Autonomic Symptom Profile (ASP)

This questionnaire is a validated self-reporting questionnaire that comprehensively evaluates autonomic symptoms on 11 subscales and produces a composite autonomic symptom score [14]. The Composite Autonomic Symptom Score (COMPASS-31) is a shortened and validated version, and it was used to assess participants. It comprises of 72 questions (orthostatic intolerance, 9 items; secretomotor, 8 items; male sexual dysfunction, 8 items; urinary dysfunction, 3 items; gastro-paresis, 5 items; constipation, 4 items; diarrhea, 4 items; pupillomotor, 7 items; vasomotor, 11 items; reflex syncope, 5 items; and sleep, 8 items) and an additional 12 items to generate 2 validity scores (an understatement index comprising of 6 questions and a psychosomatic index comprising of 6 questions). It has been validated and used extensively [14].

#### 2.5.2. The GODIN Questionnaire

This questionnaire is used to evaluate the level of activity performed in free time. It has two questions, with the first evaluating the number of times, on average, that the patient performs strenuous, moderate and mild/light exercise for more than 15 min in their free time in a typical week (7-day period) [15].

The leisure scores can be used to rank people from lowest to highest physical activity levels.

Additionally, the moderate and strenuous physical activity scores obtained can be used to classify the respondents into active and insufficiently active categories according to published movement guidelines.

The second question asks: “how often do you engage in regular activity long enough that it causes you to sweat (rapid heartbeat) during a typical week (7-day period)?”.

The score summarizes the points given to strenuous, moderate and mild/light physical activity, it is 9, 5 and 3, respectively. The overall score is between 0 and 119, with the higher scores indicating more physical activity [16]. This questionnaire has shown valid evidence in classifying subjects into active and insufficiently active categories [17].

#### 2.5.3. Pain Catastrophizing Scale

This is a validated questionnaire where the mechanism by which the pain experience is affected by catastrophism is evaluated. It was first developed in 1995 and contains 3 different aspects. The first part, called “helplessness”, corresponds to questions 1 to 5 and 12 and refers to what the person believes they have been able to do to influence their pain [5].

The second part called “magnification” corresponds to questions 6, 7 and 13 and refers to the exaggeration of the threatening properties of the painful stimulus.

Lastly, “rumination” corresponds to questions 8 to 11 and refers to the fact that the patient is unable to stop thinking about the pain and cannot get away from the idea [5].

Therefore, the questionnaire consists of 13 items divided into three subsections. The scoring scale is from 1 to 5 with the final scores ranging from 0 to 52. Higher scores correspond to higher levels of catastrophism [5].

#### 2.5.4. Tampa Kinesiophobia Scale (TSK-11)

The Spanish version was used. It measures the fear a patient has to movement. It consists of 11 items each with 1 of 4 response options, where “strongly disagree” scores 1 point and “strongly agree” scores 4 points. Hence, the total score will vary between a minimum of 11 and a maximum of 44. A high score translates as a greater fear of movement/injury, that is, high levels of Kinesiophobia [5,18]. The Tampa Scale for Kinesiophobia-11 has been shown to be consistent, reliable, and appropriate to assess fear of movement in patients with FM within a clinical context [19]. 

#### 2.5.5. Self-Efficacy Scale

This questionnaire evaluates the personal confidence a person has to carry out an activity with the aim of being successful, that is, the perception a person has of their competence to handle a stressful situation efficiently. It consists of 10 items with a response scale of four points [5,20]. The final score ranges from 0 to 44, with higher scores meaning higher perception of competence to handle a stressful situation efficiently. The Self-efficacy scale has shown adequate psychometric properties and is considered to be a useful tool in helping health professionals monitor patients’ self-efficacy perception and programming both physical activity and walking exercise intervention goals and their implementation [21].

### 2.6. Data Management

To preserve the confidentiality of the data, the study participants were assigned an identification number that they kept during the study. A list of participant identification numbers was created and separated from disidentified data. 

Statistical analyses were performed using the identification numbers in order to keep the participants anonymous and the statistician was blinded. 

### 2.7. Statistical Analysis

The statistical analysis was performed using SPSS® Statistics version 21.0 (IBM, Chicago, IL, USA), for Windows. The Kolmogorov-Smirnov test was used to evaluate the normality of the sample.

Subsequently, the descriptive statistics for the total sample, as a whole, were calculated. This included measurements of central tendency and dispersion ranges using the mean and standard deviation (SD) to describe parametric ranges, and the skewness and kurtosis to describe the data. Finally, correlations were made between the different variables using Pearson correlation coefficients since all variables followed a normal distribution. A weak correlation was defined as values between 0.3 and 0.5; moderate between 0.5 and 0.7; and strong if the correlation coefficient was more than 0.7. A *p*-value < 0.05 was considered statistically significant [22].

Based on results of other randomised clinical trials and previous reviews [23,24], a minimum of 22 patients per group is needed to obtain a statistical power of 90%, with a value of α = 0.05 and the standard deviation of the NPRS of 2.0 units to detect this difference between subjects. The final sample of the study was 41 women with FM.

## 3. Results

Demographic characteristics are shown in Table 1. There were no significant differences between variables within the group. 

No association was found between the level of daily physical activity (measured by the Godin Leisure questionnaire) and vegetative symptoms, nor with any psychological factor studied (Table 2). However, a low and significant level of association was found between pain catastrophizing (PCS) and Kinesiophobia (r = 0.398; *p* < 0.01), as well as with catastrophizing and vegetative symptoms (r = 0.428; *p* < 0.05). Furthermore, a low and significant level of association was also found between self-efficacy and vegetative symptoms (r = 0.397; *p* < 0.05).

## 4. Discussion

The objective of the paper was to study the level of association between the amount of daily physical activity, psychological factors (catastrophism, self-efficacy and fear of movement) and vegetative symptoms in patients with fibromyalgia. No association was found between the level of daily physical activity (measured by the Godin Leisure questionnaire) and vegetative symptoms, nor with any psychological factor studied. However, a low and significant level of association was found between pain catastrophizing (PCS) and Kinesiophobia (r = 0.398; *p* < 0.01), as well as with PCS and vegetative symptoms (r = 0.428; *p* < 0.05). Furthermore, a low and significant level of association was found between self-efficacy and vegetative symptoms (r = 0.397; *p* < 0.05).

Current evidence considers fibromyalgia to be a multidimensional pathology, however, there are few studies that analyze different components in the same study [25]. In regard to the level of physical activity, there is no consensus or certainty about what type of exercise is the most optimal for health and the reduction in symptoms in patients with fibromyalgia (FM), both in the short term and especially in the long term [26]. Currently, low and medium intensity exercise seems to improve the quality of life in patients with FM [27], however, some authors consider high intensity exercise to be just as beneficial for their health [28]. With regards to pain, strength exercise may be the most indicated, while aerobic exercise would improve quality of life. Thus, the combination of the aforementioned exercise modalities should be the most beneficial for patients with FM [2].

The benefits of physical activity are widely known. However, determining its frequency and intensity in different populations in general, and in the FM population in particular, with the aim of improving quality of life and pain, along with psychosocial factors, remains an outstanding goal. Our results showed no association between the amount of daily physical activity and psychological factors or vegetative symptoms in patients with FM, nevertheless other authors did find such an association [29]. The tool used to assess pain may explain this association. While Galvez Sanchez et al. determined pain through the McGill questionnaire (severity of pain), our study used a questionnaire focused on the symptoms of vegetative origin, which is currently considered to be the key to understanding the etiology of FM given its central involvement [30]. However, our study found an association between pain catastrophizing and kinesiophobia (r = 0.398; *p* = 0.01), as well as an association with vegetative symptoms (r = 0.428; *p* = 0.005), which is in line with other studies. Along these lines, Bruna et al. showed a relationship between kinesiophobia and disability in patients with FM. Other studies focused on stress as the key factor influencing and mediating the different psychological factors in patients with FM, although this information is not comparable with our study, since it did not measure any variable in relation to stress [31]. 

The psychosocial factors and vegetative symptoms, resulting from the alteration of the central nervous system, are one of the mechanisms by which the symptoms of FM patients are explained. In this context, in a recent review with meta-analysis, Schütze et al. showed that different types of intervention techniques are capable of reducing pain catastrophism, but appraisal of these changes indicated only a modest effect [32]. Surprisingly, the results showed that other types of interventions, such as physical exercise or the combination of cognitive behavioral therapy (CBT) and physical exercise, also reduced the symptoms suggesting that a treatment with such heterogeneity makes it difficult to show conclusive results [33].

In the fear-avoidance model, catastrophizing was conceptualized as a key cognitive element in generating fear and avoidance behaviors. In fact, catastrophizing was identified as the turning point at which individuals enter or do not enter the cycle of fear avoidance. The cycle starts if the pain is misinterpreted as a catastrophe. The catastrophic misinterpretations of pain lead to an extreme fear of experiencing more pain or being (re)injured, which progressively extends to a fear of physical movement and eventually to a total avoidance of movement and activity. The long-term avoidance of physical activity has several consequences, from impaired functioning and physical debilitation of the individual, to increased negative mood that contributes to creating a psychological sense of disability, which if prolonged can lead to depression [34,35].

Chronic pain represents a constant vigilance to the threat of pain leading to persisting attempts to escape from pain. The prolonged experience of inescapable pain generates a high level of awareness about one’s own body, a great difficulty in disconnecting from pain, and high levels of symptom reporting [36]. In this vein, a very recent study shows how patients with chronic musculoskeletal pain present significant disturbances in the circadian variation of blood pressure, which may be the result of the aforementioned ideas, showing an alteration of the HPA axis, biorhythm, and autonomic nervous system, leading again to the perpetuation of pain [10].

Therefore, the results obtained may have clinical implications, since psychological factors may influence not only pain perception as it is known, but also the presence of vegetative symptoms. Fear induces a fight/flight reaction in which the parasympathetic nervous system is inhibited, leaving the sympathetic system without opposition and causing vegetative symptoms as a consequence [37]. Association between multiple factors in people suffering from complex diseases such as fibromyalgia is further explained by the law of Donald Hebb: “Neurons that fire together, wire together”. However, the underlying mechanisms were not assessed, and future research should shed light on this, in line with the known emotional motor system described by Holstege [13]. In this context, psychological factors and specifically fear and the perception of threat have been seen to alter activities of the pelvic organs, such as micturition, defecation, and sexual activities, which can only take place when the situation is safe. Furthermore, other functions such as heart rate, vocalization, respiration, and blood pressure, may also be affected [13]. Therefore, the presented results may support the need for cognitive therapies as part of the treatment for those suffering from FM.

On the other hand, depression could also have had an influence on the perception of pain and vegetative symptoms. However, we did not evaluate depression in the participants of our study, and this should be investigated.

Other factors such as microbiota, immune function, and potential persistent pathogens should be also discussed when understanding the relationship between psychological factors and vegetative symptoms in subjects with FM. In this regard, the gut microbiota is thought to play a role in FM when vegetative symptoms occur, as in irritable bowel syndrome, likely changing the existing microbiome not only in the gastrointestinal system but also within the emerging field of the gut-musculoskeletal axis [38]. Interestingly, some studies have shown *Helicobacter pylori* to be significantly increased in those with FM [38], as well as altered levels of urine metabolites and gut bacteria (r Intestinimonas butyriciproducens, Flavonifractor plautii, Butyricoccus desmolans, Eisenbergiella tayi and Eisenbergiella massiliensis) [39], with the presence of the monosaccharides sorbose, rhamnose, and tagatose which are typically not found in human urine. Olama et al., described significant associations between *H. pylori* immunoglobulin-G (IgG)-positive patients and disease markers, including post-exertion pain, morning stiffness, confusion, mood, tension headache, sleep disturbance, dyscognition, changes in appetite and fatigue (all *p* < 0.05) [40]. Gezici’s group reported a significant reduction in pain, as evaluated by the number of tender points, following *H. pylori* eradication (*p* < 0.001) [41]. Furthermore, changes in other bacterial populations such as Bifidobacterium, Lactobacilus, Akkermansia muciniphila, and Parabacteroides should be further studied, since a relationship between them and higher levels of glutamate has been shown, which affects the central nervous system and therefore pain signaling, the appearance of potential vegetative symptoms and finally impaired psychological health, being depression and autism the most studied disorders [38,42]. This context may lead to changes in mitochondrial function in immune and glial cells via gut dysbiosis and increased gut permeability. The increase in lipopolysaccharides and HMGB1 and the decrease in gut microbiome-derived butyrate dysregulate mitochondrial function both directly and via increased ceramide and decreased melatonin and vagal function. Then, dysregulation in circadian rhythms arises, importantly altering the re-setting of mitochondrial function in immune and glial cells over the circadian rhythm, as has been shown in subjects suffering from chronic fatigue syndrome [43].

The strengths of this study should be highlighted. First and foremost, it takes into account different fundamental factors when evaluating and understanding FM, such as physical activity, important psychological factors such as self-efficacy, catastrophism and fear of pain, in addition to vegetative symptoms, which consider the alteration of the central nervous system as part of the etiology of FM, as well as discuss potential interactions between gut microbiome, mitochondrial and immune functions in subjects with FM. The results may be clinically useful and propose new avenues of study. However, some weaknesses must be recognized. The study design is cross-sectional, therefore, the results must be interpreted with caution, as must their extrapolation. The study sample is small, and there are differences in some collected variables, such as weight. Other tools such as accelerometers could have been used to assess physical activity, and potential bias related to self-reported measures, as well as the potentially low reliability of scales must be acknowledged.

On the other hand, our results may have clinical application and use, since the association between vegetative symptoms, pain catastrophizing and kinesiophobia suggests taking these psychological factors into account when preparing a treatment program for FM patients. In addition, incorporating other biomarkers, such as cortisol and melatonin, to assess patient stress and circadian rhythms, the composition of the gastrointestinal tract microbiota, its metabolites, luminal neurotransmitters and brain neurochemistry in addition to nutritional and lifestyle factors in people with fibromyalgia, would be necessary. Finally, it is necessary to determine what type of physical activity most influences patients with fibromyalgia, together with the most optimal time of day to carry it out, as well as the addition of interventions focused on improving mitochondrial health.

## 5. Conclusions

An association between vegetative symptoms, pain catastrophizing and kinesiophobia has been shown in patients with fibromyalgia. Future studies should further verify our findings and shed light on these and other underlying mechanisms, together with already known treatments, which may open up new multi-etiological interventions at the level of nutrition and lifestyle.

## Figures and Tables

**Table 1:** Summary of sociodemographic data of the women diagnosed with Fibromyalgia.

Variable	Women Diagnosed with Fibromyalgia (*n* = 41)		Skewness	Kurtosis	K-S (*p*)
	Mean ± SD/Frequency (%)	95% CI			
**Age (years)**	52.60 ± 8.04	[50.10, 55.10]	−0.11	−0.66	0.10 (0.801)
**Height (m)**	1.63 ± 0.05	[1.62, 1.65]	−0.06	−0.77	0.09 (0.883)
**Weight (kg)**	78.19 ± 18.03	[72.57, 84.00]	0.33	−1.09	0.11 (0.623)
**BMI (kg/m^2^)**	29.37 ± 6.33	[27.37, 31.37]	0.33	−1.52	0.17 (0.169)
**SSS**	8.51 ± 1.31	[8.10, 8.92]	0.36	−0.58	0.12 (0.562)
**WPI**	8.17 ± 2.54	[7.37, 8.97]	0.30	−0.64	0.13 (0.119)
**Years of diagnosed FM**	8.98 ± 2.78	[8.10, 9.85]	0.16	0.59	0.11 (0.696)
**Godin Leisure**	29.10 ± 18.23	[23.34, 34.85]	0.08	−0.82	0.11 (0.647)
**PCS**	27.12 ± 12.62	[23.14, 31.11]	−0.26	−1.16	0.10 (0.730)
**Self-Efficacy**	27.54 ± 4.84	[26.01, 29.06]	0.04	−0.44	0.09 (0.915)
**TSK-11**	27.51 ± 6.87	[25.34, 29.68]	−0.51	−0.58	0.14 (0.380)
**Compass-31**	44.58 ± 18.22	[38.83, 50.33]	0.35	0.13	0.13 (0.517)

Note. Data are expressed as mean ± SD for quantitative variables and as frequency (%) for qualitative variables. Abbreviations: CI (confidence interval); BMI (body mass index); SSS (Symptom Severy Score); WPI (Widespread Pain Index); PCS (Pain Catastrophizing Scale); TSK (Tampa Scale Kinesiophobia); K-S (Kolmogorov-Smirnov).

**Table 2:** Matrix correlation between variables.

		Godin Leisure	PCS	Self-Efficacy	TSK	Compass-31
Godin leisure	R de Pearson	-				
	IC 95% Superior	-				
	IC 95% Inferior	-				
PCS	R de Pearson	0.268	-			
	IC 95% Superior	0.532	-			
	IC 95% Inferior	−0.043	-			
Self-Efficacy	R de Pearson	0.023	0.275	-		
	IC 95% Superior	0.328	0.537	-		
	IC 95% Inferior	−0.287	−0.036	-		
TSK	R de Pearson	−0.059	0.398 **	0.115	-	
	IC 95% Superior	0.253	0.629	0.408	-	
	IC 95% Inferior	−0.360	0.103	−0.200	-	
Compass-31	R de Pearson	−0.003	0.428 **	0.397 *	0.056	-
	IC 95% Superior	0.305	0.650	0.628	0.357	-
	IC 95% Inferior	−0.311	0.138	0.102	−0.256	-

Note. * *p* < 0.05, ** *p* < 0.01. PCS (Pain Catastrophizing Scale); TSK (Tampa Scale Kinesiophobia).

## Data Availability

Not applicable.

## Abbreviations

ASP	Autonomic Symptom Profile
FM	Fibromyalgia
HPA	Hypothalamic-pituitary axis
PPT	Pain pressure threshold
TSK	Tampa Scale Kinesiophobia
CBT	cognitive behavioral therapy

## References

[1] Lorente L.C., Ríos M.C.G., Ledesma S.N., Haro R.M.T., Barragán A.C., Correa-Rodríguez M., Ferrándiz M.E.A. (2019). Functional Status and Body Mass Index in Postmenopausal Women with Fibromyalgia: A Case–Control Study. Int. J. Environ. Res. Public Health.

[2] Sosa-Reina M.D., Nunez-Nagy S., Gallego-Izquierdo T., Pecos-Martín D., Monserrat J., Álvarez-Mon M. (2017). Effectiveness of Therapeutic Exercise in Fibromyalgia Syndrome: A Systematic Review and Meta-Analysis of Randomized Clinical Trials. Biomed. Res. Int..

[3] Salgueiro M., Buesa I., Aira Z., Montoya P., Bilbao J., Azkue J.J. (2009). Valoración de Factores Sociales y Clínicos En El Sindrome de Fibromialgia. Rev. Soc. Española Dolor.

[4] Kulshreshtha P., Deepak K.K. (2013). Autonomic Nervous System Profile in Fibromyalgia Patients and Its Modulation by Exercise: A Mini Review. Clin. Physiol. Funct. Imaging.

[5] Navarro-Ledesma S., Gonzalez-Muñoz A., Carroll J., Burton P. (2022). Short- and Long-Term Effects of Whole-Body Photobiomodulation on Pain, Functionality, Tissue Quality, Central Sensitisation and Psychological Factors in a Population Suffering from Fibromyalgia: Protocol for a Triple-Blinded Randomised Clinical Trial. Ther. Adv. Chronic Dis..

[6] Wolfe F., Clauw D.J., Fitzcharles M.A., Goldenberg D.L., Häuser W., Katz R.L., Mease P.J., Russell A.S., Russell I.J., Walitt B. (2016). 2016 Revisions to the 2010/2011 Fibromyalgia Diagnostic Criteria. Semin. Arthritis Rheum..

[7] Mas A.J., Carmona L., Valverde M., Ribas B., Navarro F., Ortiz A.M., Ribas B., Rojas P., Rodríguez-Lozano C., Romero F. (2008). Prevalence and Impact of Fibromyalgia on Function and Quality of Life in Individuals from the General Population: Results from a Natiowide Study in Spain. Clin. Exp. Rheumatol..

[8] Queiroz L.P. (2013). Worldwide Epidemiology of Fibromyalgia. Curr. Pain Headache Rep..

[9] Vos T., Abajobir A.A., Abbafati C., Abbas K.M., Abate K.H., Abd-Allah F., Abdulle A.M., Abebo T.A., Abera S.F., Aboyans V. (2017). Global, Regional, and National Incidence, Prevalence, and Years Lived with Disability for 328 Diseases and Injuries for 195 Countries, 1990-2016: A Systematic Analysis for the Global Burden of Disease Study 2016. Lancet.

[10] Navarro-Ledesma S., Gonzalez-Muñoz A., Garcia-Rios M.C., de la Serna D., Pruimboom L. (2022). Circadian Variation of Blood Pressure in Patients with Chronic Musculoskeletal Pain: A Cross-Sectional Study. Int. J. Environ. Res. Public Health.

[11] Thornton K.G.S., Robert M. (2019). Prevalence of Pelvic Floor Disorders in the Fibromyalgia Population: A Systematic Review. J. Obstet. Gynaecol. Can..

[12] Kortlever J.T.P., Janssen S.J. (2015). What Is the Most Useful Questionnaire for Measurement of Coping Strategies in Response to Nociception?. Clin. Orthop. Relat. Res..

[13] Holstege G. (2016). How the Emotional Motor System Controls the Pelvic Organs. Sex. Med. Rev..

[14] Sletten D.M., Suarez G.A., Low P.A., Mandrekar J., Singer W. (2012). COMPASS 31: A Refined and Abbreviated Composite Autonomic Symptom Score. Mayo Clin. Proc..

[15] Fattahi M.R., Noormohammadpour P., Ramezani M., Mesgarof M.A., Abolhasani M. (2021). Translation and Validation of the Persian Version of Godin Leisure-Time Exercise Questionnaire in Patients with Multiple Sclerosis. BMC Neurol..

[16] Amireault S., Godin G., Lacombe J., Sabiston C.M. (2015). The Use of the Godin-Shephard Leisure-Time Physical Activity Questionnaire in Oncology Research: A Systematic Review. BMC Med. Res. Methodol..

[17] Amireault S., Godin G. (2015). The Godin-Shephard Leisure-Time Physical Activity Questionnaire: Validity Evidence Supporting Its Use for Classifying Healthy Adults into Active and Insufficiently Active Categories. Percept. Mot. Ski..

[18] Larsson C., Hansson E.E., Sundquist K., Jakobsson U. (2016). Kinesiophobia and Its Relation to Pain Characteristics and Cognitive Affective Variables in Older Adults with Chronic Pain. BMC Geriatr..

[19] Salvador E.M.E.S., Franco K.F.M., Miyamoto G.C., Franco Y.R.d.S., Cabral C.M.N. (2021). Analysis of the Measurement Properties of the Brazilian-Portuguese Version of the Tampa Scale for Kinesiophobia-11 in Patients with Fibromyalgia. Braz. J. Phys. Ther..

[20] Jurado M.d.M.M., Pérez-Fuentes M.d.C., Ruiz N.F.O., Márquez M.d.M.S., Linares J.J.G. (2019). Self-Efficacy and Emotional Intelligence as Predictors of Perceived Stress in Nursing Professionals. Medicina.

[21] López-Roig S., Pastor-Mira M.Á., Núñez R., Nardi A., Ivorra S., León E., Peñacoba C. (2021). Assessing Self-Efficacy for Physical Activity and Walking Exercise in Women with Fibromyalgia. Pain Manag. Nurs..

[22] Mukaka M.M. (2012). Statistics Corner: A Guide to Appropriate Use of Correlation Coefficient in Medical Research. Malawi Med. J..

[23] Dworkin R.H., Turk D.C., Wyrwich K.W., Beaton D., Cleeland C.S., Farrar J.T., Haythornthwaite J.A., Jensen M.P., Kerns R.D., Ader D.N. (2008). Interpreting the Clinical Importance of Treatment Outcomes in Chronic Pain Clinical Trials: IMMPACT Recommendations. J. Pain.

[24] Eldridge S.M., Chan C.L., Campbell M.J., Bond C.M., Hopewell S., Thabane L., Lancaster G.A., O’Cathain A., Altman D., Bretz F. (2016). CONSORT 2010 Statement: Extension to Randomised Pilot and Feasibility Trials. Pilot Feasibility Stud.

[25] Borchers A.T., Gershwin M.E. (2015). Fibromyalgia: A Critical and Comprehensive Review. Clin. Rev. Allergy Immunol..

[26] Busch A.J., Webber S.C., Brachaniec M., Bidonde J., Bello-Haas V.D., Danyliw A.D., Overend T.J., Richards R.S., Sawant A., Schachter C.L. (2011). Exercise Therapy for Fibromyalgia. Curr. Pain Headache Rep..

[27] Bidonde J., Busch A.J., Schachter C.L., Overend T.J., Kim S.Y., Góes S.M., Boden C., Foulds H.J.A. (2017). Aerobic Exercise Training for Adults with Fibromyalgia. Cochrane Database Syst. Rev..

[28] Atan T., Karavelioğlu Y. (2020). Effectiveness of High-Intensity Interval Training vs. Moderate-Intensity Continuous Training in Patients with Fibromyalgia: A Pilot Randomized Controlled Trial. Arch. Phys. Med. Rehabil..

[29] Galvez-Sánchez C.M., Reyes del Paso G.A., Duschek S. (2018). Cognitive Impairments in Fibromyalgia Syndrome: Associations with Positive and Negative Affect, Alexithymia, Pain Catastrophizing and Self-Esteem. Front. Psychol..

[30] Maugars Y., Berthelot J.M., Le Goff B., Darrieutort-Laffite C. (2021). Fibromyalgia and Associated Disorders: From Pain to Chronic Suffering, From Subjective Hypersensitivity to Hypersensitivity Syndrome. Front. Med..

[31] Malin K., Littlejohn G.O. (2016). Psychological Factors Mediate Key Symptoms of Fibromyalgia through Their Influence on Stress. Clin. Rheumatol..

[32] Schütze R., Rees C., Smith A., Slater H., Campbell J.M., O’Sullivan P. (2018). How Can We Best Reduce Pain Catastrophizing in Adults with Chronic Noncancer Pain? A Systematic Review and Meta-Analysis. J. Pain.

[33] Petrini L., Arendt-Nielsen L. (2020). Understanding Pain Catastrophizing: Putting Pieces Together. Front. Psychol..

[34] Vlaeyen J.W.S., Crombez G., Linton S.J. (2016). The Fear-Avoidance Model of Pain. Pain.

[35] Aldrich S., Eccleston C., Crombez G. (2000). Worrying about Chronic Pain: Vigilance to Threat and Misdirected Problem Solving. Behav. Res. Ther..

[36] Van Damme S., Legrain V., Vogt J., Crombez G. (2010). Keeping Pain in Mind: A Motivational Account of Attention to Pain. Neurosci. Biobehav. Rev..

[37] Karsenty G. (2020). That Feeling in Your Bones.

[38] Erdrich S., Hawrelak J.A., Myers S.P., Harnett J.E. (2020). Determining the Association between Fibromyalgia, the Gut Microbiome and Its Biomarkers: A Systematic Review. BMC Musculoskelet. Disord..

[39] Minerbi A., Fitzcharles M.-A. (2020). Gut Microbiome Pertinence in Fibromyalgia. Clin. Exp. Rheumatol..

[40] Olama S.M., El-Arman M. (2013). Helicobacter Pylori in Egyptian Patients with Fibromyalgia Syndrome. Egypt. Rheumatol..

[41] Gezici E., Alpayci M., Özkan Y., Küçük M.E., Ünver H., Hiz Ö. (2014). The Effects of Helicobacter Pylori Eradication on the Number of Tender Points, Sleep Quality, Depression, and Anxiety in Patients with Fibromyalgia. Arch. Rheumatol..

[42] Clos-Garcia M., Andrés-Marin N., Fernández-Eulate G., Abecia L., Lavín J.L., van Liempd S., Cabrera D., Royo F., Valero A., Errazquin N. (2019). Gut Microbiome and Serum Metabolome Analyses Identify Molecular Biomarkers and Altered Glutamate Metabolism in Fibromyalgia. EBioMedicine.

[43] Anderson G., Maes M. (2020). Mitochondria and Immunity in Chronic Fatigue Syndrome. Prog. Neuro-Psychopharmacol. Biol. Psychiatry.

